# A Green Approach to Bio-Based Active Packaging: Grape Skin Extract-Synthesized AgNPs for Food Preservation

**DOI:** 10.3390/ma19020218

**Published:** 2026-01-06

**Authors:** Wenjia Yin, Yongzhen Lei, Jiayi Wang, Qin Lei, Wenxi Yu, Siyu Ou

**Affiliations:** 1 School of Packaging Design Art, Hunan University of Technology, Zhuzhou 412007, China; m23135108093@stu.hut.edu.cn (W.Y.); m24135700060@stu.hut.edu.cn (J.W.); 2 School of Packaging Engineering, Hunan University of Technology, Zhuzhou 412007, China; m230805z1003@stu.hut.edu.cn (Q.L.); yuwx@hut.edu.cn (W.Y.); m24080500009@stu.hut.edu.cn (S.O.)

**Keywords:** sliver nanoparticles, polyvinyl alcohol, chitosan, antimicrobial film, food preservation

## Abstract

This study aimed to develop an environmentally friendly composite film with effective antibacterial and preservation properties. Silver nanoparticles (AgNPs) were green-synthesized using grape skin extract as a natural reducing agent and incorporated into a PVA/chitosan matrix. The composition of the extract and the structural characteristics of the AgNPs were characterized by UPLC-MS and TEM. The barrier, mechanical performance, antibacterial, and fruit preservation properties of the resulting films were systematically evaluated. The results showed that the incorporation of AgNPs significantly improved the water vapor and oxygen barrier properties of the film and imparted excellent broad-spectrum antibacterial activity. In grape storage experiments, films with higher AgNPs content effectively delayed skin aging and moisture loss, maintaining better visual quality of the fruit. This work provides a green and feasible approach for the preparation of nanoparticle-enhanced antibacterial packaging materials based on natural products, with promising application potential.

## 1. Introduction

Grapes are a nutritionally rich fruit, yet they are highly perishable due to water loss [1], microbial contamination [2,3], and oxidative reactions [4], all of which shorten their postharvest life. Conventional preservation methods such as chemical preservatives and wax coatings can mitigate deterioration to some degree, but they may compromise flavor quality and raise concerns about consumer health and safety [5,6,7]. As a result, there has been increasing interest in green and eco-friendly packaging strategies, particularly edible coatings and biodegradable films, to extend the shelf life of grapes and other fresh produce.

Traditional packaging materials offer limited inhibition of microbial growth, which has driven the development of active and antibacterial packaging systems [8,9,10,11] designed to reduce foodborne disease risks and maintain food quality. Among emerging nanomaterials, silver nanoparticles (AgNPs) have attracted special attention due to their high stability, heat resistance, and strong antimicrobial activity [12]. Numerous studies have demonstrated that AgNPs effectively suppress bacterial growth and biofilm formation, thereby significantly prolonging food shelf life [13,14,15,16]. The antibacterial mechanism of AgNPs is generally attributed to their ability to attach to bacterial cell membranes, generate reactive oxygen species (ROS), and release Ag^+^ ions, which disrupt cellular respiration, damage DNA, and ultimately cause cell death. These unique properties make AgNPs highly promising for food preservation and biomedical applications.

Within polymeric matrices, polyvinyl alcohol (PVA) is widely recognized as a sustainable packaging material due to its excellent film-forming properties, biocompatibility, and biodegradability [17,18]. To enhance its functionality, PVA is often blended with natural polysaccharides such as chitosan (CS), resulting in composite films with superior mechanical strength, barrier properties, and antibacterial potential [19,20].

At the same time, attention has shifted toward green synthesis of AgNPs to avoid hazardous chemical reductants. Plant extracts rich in phenolic compounds and flavonoids have been extensively employed as natural reducing and capping agents. For example, marigold flower extracts have been used to produce AgNPs with nematicidal activity against root-knot nematodes [21]; guava leaf extracts yielded AgNPs with antifungal properties against chili fruit rot pathogens [22]; and Aloe vera-derived AgNPs, combined with Ampelopsis brevipedunculata extracts, were integrated into nanofibrous scaffolds to accelerate wound healing in vivo [23]. PEG-assisted systems have demonstrated long-term nanoparticle stability, while grape seed extracts have been utilized to prepare AgNPs with excellent catalytic and antimicrobial activities [24,25]. Despite these advances, most existing studies focus on leaves, seeds, or medicinal plants, while grape skins, an abundant by-product of winemaking and juice processing, remain underexplored.

While previous research has explored the valorization of grape by-products for nanoparticle synthesis, the development of AgNPs-based packaging films, and the application of such films in food preservation, these aspects have often been investigated in isolation. The present work distinguishes itself by integrating these strands into a focused and coherent study that explicitly bridges agricultural waste valorization with targeted food preservation. Unlike studies that primarily utilize grape seeds or focus on non-food applications, this research specifically employs grape skin extract (GSE)—an underutilized, phytochemically rich waste material—as a green synthesis platform for AgNPs. Furthermore, our approach advances the field by not only fabricating a nanocomposite film but also by establishing a clear structure–property–application relationship. This is achieved through a detailed phytochemical analysis of GSE to elucidate the reduction mechanism, a systematic investigation of how a concentration gradient (2–8 wt%) of GSE-synthesized AgNPs modulates film properties, and a direct efficacy evaluation for preserving the visual and sensory quality of fresh table grapes—a highly perishable commodity. Thus, this study carves out a specific niche by offering a complete, analytically grounded “waste-to-protection” strategy for a defined application.

Therefore, the aim of this study is to fabricate and characterize novel PVA-/CS-based antibacterial films incorporated with GSE-synthesized AgNPs. The films were prepared via a casting method using glycerol as a plasticizer, and their structural, mechanical, barrier, and antimicrobial properties were systematically evaluated. Beyond demonstrating an eco-friendly synthesis route, this work proves that grape skin-mediated AgNPs can be effectively integrated into biodegradable films to provide robust antimicrobial protection, thereby offering a practical solution for extending the shelf life of fresh grapes and enhancing food safety.

## 2. Materials and Methods

### 2.1. Reagents and Materials

Fresh grapes were purchased at the school supermarket. Polyvinyl alcohol (PVA, AR grade), acetic acid (AR grade), and glycerol (AR grade) were procured from Tianjin Damao Chemical Reagent Factory (Tianjin, China). Chitosan (food grade) was purchased from Sinopharm Group Co., Ltd. (Shanghai, China). Silver nitrate (AgNO_3_, AR grade) was provided by Shenzhen Bolinda Technology Co., Ltd. (Shenzhen, China). All experimental solutions were prepared using purified water (Nongfu Spring, China Resources Nongfu Spring Beverage Co., Ltd., Hangzhou, China).

### 2.2. Preparation of Grape Skin Extract (GSE)

The grape skin extract (GSE) was prepared through a two-stage process of pretreatment and aqueous extraction. Initially, fresh grape skins, obtained by manually peeling washed grapes, were dehydrated in a forced-air oven at 60 °C for 10–12 h. The dried skins were then pulverized into a fine powder using a grinder and passed through a 100-mesh sieve. For extraction, 1.0 g of the powdered material was precisely weighed and combined with 30 mL of distilled water. This mixture was subjected to heating at 65 °C for 1.5 h. The cooled extract was filtered and employed directly for subsequent UPLC-MS compositional analysis and as the green reagent for AgNPs synthesis. Visual documentation of the key stages in grape processing is provided in Figure S1.

### 2.3. UHPLC-QTOF-MS/MS Analysis

The phytochemical composition of GSE was profiled using a UHPLC-QTOF-MS/MS system. A QTOF X500B mass spectrometer (SCIEX, Framingham, MA, USA) with an Exion AD UPLC system was used for sample analysis. Chromatographic separation was carried out on an HSS T3 column (2.1 × 100 mm, 1.8 μm) maintained at 40 °C. A binary mobile phase of (A) water with 0.05% formic acid and (B) acetonitrile with 0.05% formic acid was employed. A 42 min linear gradient from 5% to 98% B was run at a flow rate of 0.3 mL/min, with a 2 μL injection volume. The autosampler temperature was set at 4 °C. For mass spectrometric detection, electrospray ionization (ESI) in positive ion mode was used with the following parameters: spray voltage, 5500 V; curtain gas, 35 psi; ion source gas 1 (GS1) and gas 2 (GS2), 50 psi each; and ion source temperature, 500 °C.

### 2.4. Preparation of AgNPs

The green synthesis of AgNPs was conducted based on previously established plant-extract methods with modifications to suit GSE [26,27]. Specifically, the GSE was combined with an aqueous solution of silver nitrate (0.75 mM) at a fixed volume ratio of 1:3 (extract:AgNO_3_). The reaction mixture was then incubated in a temperature-controlled water bath at 85 °C for 2 h. The reduction of Ag^+^ ions was monitored visually, and the process was considered complete when the solution developed a stable reddish-brown coloration, characteristic of AgNPs formation. A schematic of the synthesis workflow is provided in Figure S2.

### 2.5. Preparation of Composite Films

The composite films were prepared via a solution casting process. First, individual polymer solutions were formulated: chitosan (CS) was dissolved in a 2% (*v*/*v*) acetic acid aqueous solution at a concentration of 0.02 g/mL, while polyvinyl alcohol (PVA) was dissolved in distilled water at 4 g per 96 mL, with the latter requiring continuous stirring at 95 °C for 2 h. Both solutions were stirred at 500 rpm using a magnetic stirrer (DF-101S, Hangzhou Jer Experimental Equipment Co., Ltd., Hangzhou, China). Then, these solutions were blended at a 1:1 mass ratio (based on polymer solids). Into this blend, a predetermined volume of the AgNPs colloid was added to achieve final loadings of 2, 4, 6, and 8 wt% relative to the total polymer mass. The mixture was homogenized by vigorous stirring at 60 °C for 2 h. Finally, the resulting homogeneous solution was cast onto glass plates, allowed to dry under ambient conditions, peeled off, and stored in a desiccator for later use. The specific compositions of all films are abbreviated and detailed in Table 1.

### 2.6. Characterization of AgNPs and Films

#### 2.6.1. Structural Characterization of AgNPs

The morphology and surface chemistry of the green-synthesized AgNPs were characterized using two complementary techniques. The size, distribution, and shape of the nanoparticles were examined by transmission electron microscopy (TEM) on a JEOL JEM-2011 instrument (JEOL Ltd., Tokyo, Japan) operated at an accelerating voltage of 200 kV, which offers a point resolution of 0.23 nm. Concurrently, the functional groups potentially involved in the reduction and stabilization of AgNPs were identified by Fourier transform infrared (FTIR) spectroscopy using a Nicolet iS10 spectrometer (Thermo Fisher Scientific, Waltham, MA, USA).

#### 2.6.2. Structural Characterization of Composite Films

The structural properties of the composite films were interrogated using multiple techniques. Fourier transform infrared (FTIR) spectra were acquired on a Nicolet iS10 spectrometer (Thermo Fisher Scientific, Waltham, MA, USA) over the range of 4000 to 400 cm^−1^ at a resolution of 4 cm^−1^. Crystalline structure was analyzed via X-ray diffraction (XRD) using a Bruker D8 Advance diffractometer (Bruker, Ettlingen, Germany) with Ni-filtered Cu Kα radiation (λ = 1.542 Å). XRD patterns were captured under operating conditions of 45 kV and 30 mA, scanning 2θ from 5° to 40° with a step size of 2° and a scan speed of 2°/min. Additionally, the surface morphology was visualized by scanning electron microscopy (SEM) on a Zeiss MIRA3LUM instrument (Carl Zeiss AG, Oberkochen, Germany), with samples observed at an accelerating voltage of 6 kV and a magnification of 5000×.

#### 2.6.3. Mechanical Properties of Composite Films

We evaluated the mechanical properties by tensile testing. Specimens (100 × 10 mm^2^) were mounted vertically in a universal testing machine (XLW(L)-P, Jinan Blu-ray Electromechanical Technology Co., Ltd., Jinan, China). Tests proceeded at a crosshead speed of 50 mm/min under controlled ambient conditions (25 °C, 50% RH). Both tensile strength (TS) and elongation at break (EAB) were recorded, with eight parallel replicates tested for each film formulation to ensure statistical reliability.

#### 2.6.4. Visible Light Transmittance

The optical properties, including color and light transmittance, were characterized to assess the films’ appearance and functionality. Film color coordinates (L, a, b) were determined with a colorimeter (10QC, Guangdong Sanenshi Intelligent Technology Co., Ltd., Guangzhou, China), calibrated against a standard background plate (L = 92.34, a* = −1.66, b* = −4.55) as per Srinivasa et al. [28], with ten measurements averaged per sample. To evaluate UV-blocking ability and transparency, film strips (10 × 40 mm^2^) were analyzed following Han et al. [29] using a UV-Vis spectrophotometer (UV-5200, Shanghai Yuan Analysis Instrument Co., Ltd., Shanghai, China), measuring transmittance at 280 nm and 600 nm.

#### 2.6.5. Barrier Performance Preparation

The Water vapor permeability (WVP) of the film was measured using water vapor transmission rate tester (C303M, Jinan Blu-ray Electromechanical Technology Co., Ltd., Jinan, China). The measurements were conducted under controlled environmental conditions of 23 °C and 90% relative humidity.

The swelling ratio (SR) is a method to evaluate the water absorption and swelling capacity of a material by measuring its mass change after immersion in a solvent. The films were cut into 40 mm × 40 mm pieces and divided into five groups (designated PC, PC_2_, PC_4_, PC_6_ and PC_8_). Each specimen was weighed to determine its initial mass. Subsequently, the specimens from each group were individually immersed in 100 mL of distilled water for 6 h. Following immersion, the specimens were removed, gently blotted to remove excess surface water, and weighed again to obtain their wet mass. The SR for each group was calculated as the ratio of the wet mass to the initial mass.

Oxygen Transmission Rate (OTR) Test was performed using a BTY-B1 gas permeability tester at 23 °C and 90% RH. Three parallel samples were measured per group, and results were averaged.

#### 2.6.6. Antimicrobial Properties

We employed the filter paper disk diffusion method [30] to evaluate the antibacterial activity of the film-forming solutions. Test organisms included *Staphylococcus aureus* (ATCC 25923) and *Escherichia coli* (ATCC 25922). Bacterial suspensions in the logarithmic growth phase were standardized to approximately 10^6^ CFU/mL. A 100 μL aliquot of each suspension was then spread evenly onto Mueller-Hinton agar plates. Sterile 6 mm filter paper disks were placed on the inoculated agar surfaces, and 10 μL of the film-forming solution (at varying concentrations) was applied to each disk. The plates, after inversion, were incubated at 37 °C for 24 h. The antibacterial efficacy was quantified by measuring and recording the diameters of the clear inhibition zones around the disks.

### 2.7. Application in Grape Preservation

**Figure 1:**
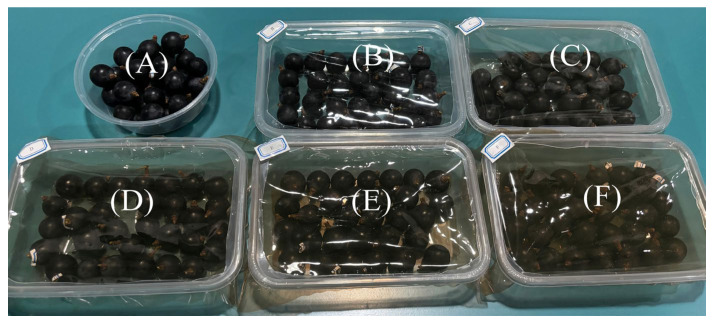
The schematic diagram of grape preservation packaging groups, which were covered by the (**A**) blank group; (**B**) PC group; (**C**) PC_2_ group; (**D**) PC_4_ group; (**E**) PC_6_ group; and (**F**) PC_8_ group.

Fresh table grapes of uniform maturity and size, free from physical defects, were selected for the preservation study. The grapes were divided into six treatment groups: a blank control, and groups treated with PC, PC2, PC4, PC6, and PC8 films, respectively. Following this grouping, each cluster was placed in a container lined with the corresponding composite film at the bottom and covered with the same film on top (see Figure 1 for packaging schematic). All samples were stored at room temperature. Throughout an 8-day storage period, the visual appearance of grapes from each group was documented and compared at designated intervals (days 1, 2, 4, 6, and 8) to evaluate the preservation effect.

## 3. Results and Discussion

### 3.1. Analysis of Plant Extract

The chemical composition of GSE was comprehensively analyzed using UPLC-MS [31], resulting in the identification of 175 compounds, including a diverse array of flavonoids, phenolic acids, organic acids, and fatty acids. Among these, 15 representative compounds are presented in the accompanying Table 2, with key constituents such as quercetin, quercitrin, rutin, isorhamnetin, and pyroglutamic acid. Notably, flavonoids exhibited significant peak areas at retention times around 10 min, indicating their predominance in the extract. Flavonoid and phenolic compounds are known for their strong reducing and stabilizing properties, enabling them to function as both reducing agents and capping agents during the biosynthesis of silver AgNPs [32]. In particular, polyhydroxylated flavonoids such as quercetin and rutin can effectively reduce Ag^+^ ions to elemental silver (Ag^0^) through their phenolic hydroxyl groups, while concurrently forming stabilizing layers on the nanoparticle surfaces [33,34]. This dual function enhances both the dispersibility and colloidal stability of the synthesized AgNPs. Moreover, the presence of fatty acids, including palmitic acid and stearic acid, may facilitate the formation of hydrophobic microenvironments during the synthesis process, promoting uniform nucleation and controlled growth of silver AgNPs [35]. The synergistic interactions among these bioactive components not only improve the efficiency of green synthesis but also endow the resulting AgNPs with potential bioactive functionalities.

### 3.2. Analysis of AgNPs and Films

#### 3.2.1. TEM Analysis of AgNPs

**Figure 2:**
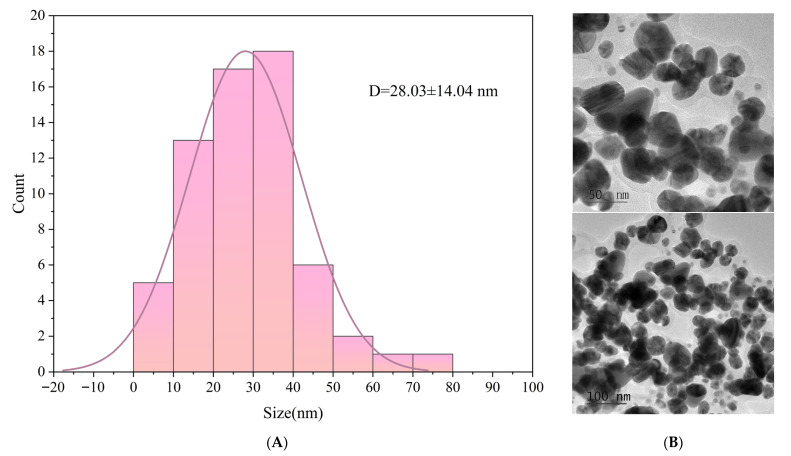
Particle size distribution (**A**); and TEM image (**B**) of AgNPs.

As shown in Figure 2, TEM analysis revealed that the silver AgNPs synthesized using GSE exhibited a predominantly spherical morphology with good dispersity and no significant aggregation. This suggests that the bioactive compounds present in the extract not only effectively reduced Ag^+^ ions to Ag^0^, but also served as stabilizing agents that capped and protected the nanoparticle surfaces. As shown in the particle size distribution analysis, the AgNPs ranged primarily from 10 to 50 nm, with an average particle size of 28.03 ± 14.04 nm. This indicates that the synthesis process was relatively mild and allowed for well-controlled nanoparticle growth.

#### 3.2.2. Microscopic Structure Analysis of Composite Films

**Figure 3:**
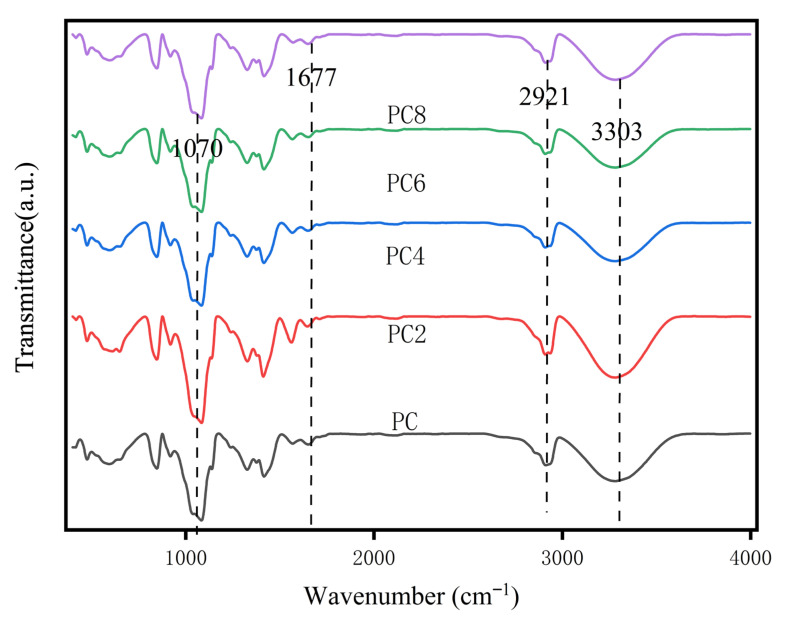
FTIR spectra of composite films.

Figure 3 shows the FTIR spectra of PVA/CS composite films (PC, PC_2_, PC_4_, PC_6_, PC_8_) with varying AgNPs content. A broad absorption band at 3300–3400 cm^−1^, assigned to –OH and –NH stretching vibrations from hydroxyl and amino groups in PVA and CS, was observed in all samples. This peak decreased in intensity and slightly red-shifted in high AgNPs content films (e.g., PC_8_), suggesting disruption of existing hydrogen bonds or formation of new hydrogen bonding or coordination interactions between AgNPs and the polymer chains. The peak at 2921 cm^−1^ corresponds to C–H stretching vibrations of methylene groups in PVA and remained nearly unchanged, indicating that the polymer backbone was not significantly affected. The peak at 1677 cm^−1^ is attributed to C=O stretching or –NH_2_ bending vibrations, mainly from residual amide or amino groups in CS. A slight shift in this peak with increasing AgNPs content suggests possible interaction between AgNPs and polar functional groups. Additionally, the absorption around 1070 cm^−1^, related to C–O–C or C–O stretching vibrations in ether and alcohol groups, was present in all samples. Variations in its intensity and shape indicate that AgNPs may influence the molecular arrangement of the polymer matrix.

**Figure 4:**
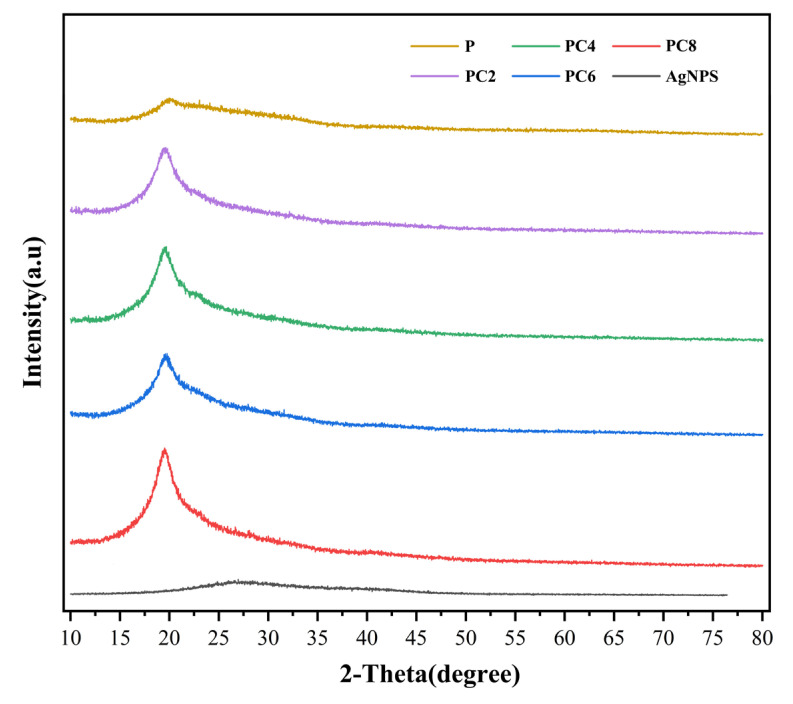
X-ray diffraction patterns for composite films.

Figure 4 shows the XRD patterns of AgNPs and a series of composite films. A broad and weak diffraction peak appears at around 2θ ≈ 19° in the pure PVA/CS film, indicating a predominantly amorphous structure with low crystallinity. Upon the incorporation of AgNPs, this characteristic peak undergoes a slight shift and a marginal increase in intensity, suggesting that the presence of AgNPs influences the arrangement and local ordering of the polymer chains. Notably, the XRD pattern of the AgNPs alone does not display the characteristic diffraction peaks of crystalline silver (e.g., the (111) and (200) planes of the face-centered cubic structure). This absence may be attributed to the small particle size, low crystallinity, and surface coverage by organic compounds derived from the plant extract used in the green synthesis process, which likely result in peak broadening or signal suppression. Furthermore, the incorporation of AgNPs into the composite films does not lead to the emergence of new crystalline peaks, indicating that the AgNPs are well dispersed within the polymer matrix and do not form large-scale crystalline aggregates.

**Figure 5:**
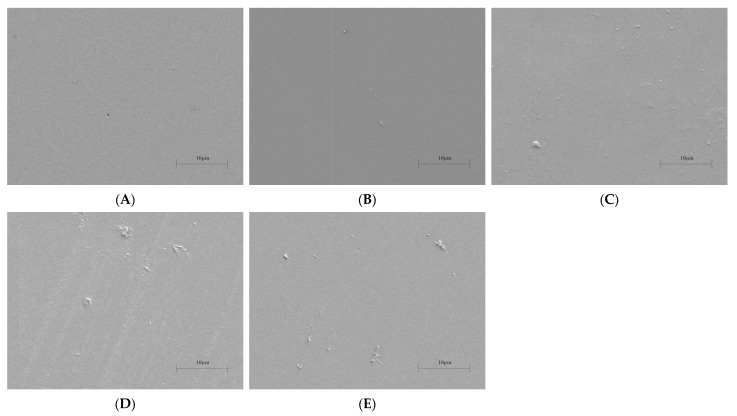
SEM images of surface of composite films: (**A**) PC film; (**B**) PC_2_ film; (**C**) PC_4_ film; (**D**) PC_6_ film, and (**E**) PC_8_ film.

Surface morphology examined by scanning electron microscopy (SEM) revealed the influence of AgNPs on film microstructure in Figure 5. While the pure PVA/CS (PC) film presented a smooth and homogeneous surface, confirming good interfacial compatibility, the incorporation of AgNPs led to progressively rougher and more irregular textures with increasing nanoparticle content. This alteration in surface topography is commonly attributed to the presence and dispersion of nanoparticles within the polymer matrix, a phenomenon consistent with observations in other PVA-based composite systems incorporating plant-synthesized AgNPs [36].

#### 3.2.3. Mechanical Properties

**Figure 6:**
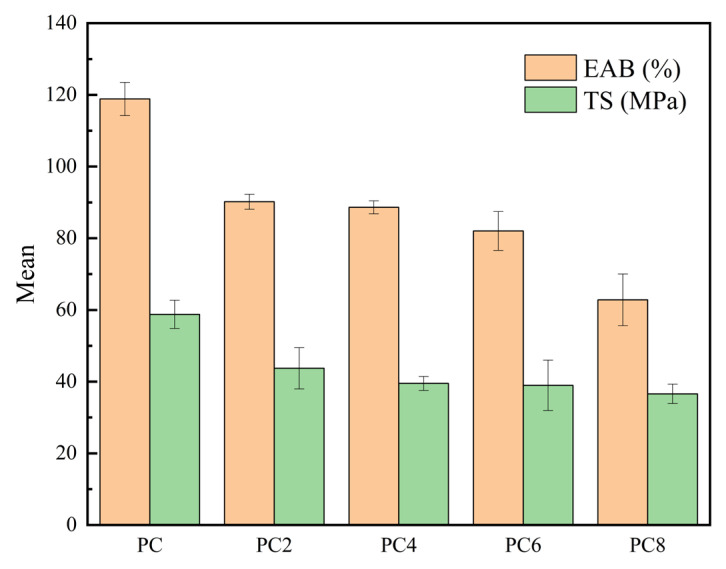
The TS and the EAB of the films.

Figure 6 illustrates the effect of varying concentrations of silver AgNPs on the mechanical properties of PVA/CS composite films. The results indicate a clear decreasing trend in both EAB and TS with increasing AgNPs content. The pure PC film exhibited the highest EAB and TS values, approximately 119% and 59 MPa, respectively. In contrast, the high AgNPs loading group (e.g., PC_8_) showed significant reductions, with EAB and TS decreasing to around 63% and 37 MPa. This reduction in mechanical performance may be attributed to nanoparticle agglomeration at higher concentrations, which disrupts the uniformity and continuity of the polymer matrix, thereby weakening stress transfer efficiency. Additionally, the introduction of AgNPs might interfere with the original hydrogen bonding and physical entanglement between PVA and CS chains, reducing chain flexibility and cooperative interactions [37].

#### 3.2.4. Measurement of Surface Color and Transmittance of Composite Films

**Figure 7:**
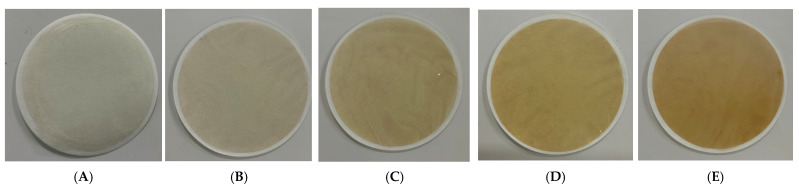
Image of composite films: (**A**) PC film; (**B**) PC_2_ film; (**C**) PC_4_ film; (**D**) PC_6_ film; and (**E**) PC_8_ film.

As shown in the visual images of the composite films in Figure 7, it can be clearly observed that the color of the films gradually darkens with increasing silver nanoparticle (AgNPs) content. Image (A), representing the PC film without AgNPs, exhibits a light grayish-white appearance with high brightness. In contrast, images (B) to (E), corresponding to PC_2_, PC_4_, PC_6_, and PC_8_ films, respectively, show a progressive deepening of color, with the PC_8_ film displaying a distinct brownish-yellow hue. This visual trend is consistent with the colorimetric data presented in Table 3, confirming the significant influence of AgNPs incorporation on the optical appearance of the films. The observed color change may be attributed to the surface plasmon resonance (SPR) effect of AgNPs, as well as their dispersion state within the polymer matrix, both of which can affect the film’s light absorption and reflection properties [38]. Overall, higher AgNPs concentrations result in darker and more uniform film coloration. This visual transformation not only indicates the successful incorporation of AgNPs but also suggests the potential applicability of these films in intelligent packaging and functional coating systems.

#### 3.2.5. Barrier Performance

Table 4 illustrates the effect of varying AgNPs content on the WVP, SR, and OTR of the composite films. The results demonstrate that the barrier properties of the films were significantly enhanced with increasing AgNPs concentration. Specifically, the WVP decreased from 60.96 for the pure PVA/CS film (PC) to 43.94 for the PC_8_ sample, indicating that the incorporation of AgNPs effectively reduced WVP ability. This improvement may be attributed to the filler effect of AgNPs within the polymer matrix, which likely resulted in a denser structure and more restricted diffusion pathways for water molecules. Similarly, the SR declined markedly from 67.35% (PC) to 24.37% (PC_8_), suggesting enhanced structural stability of the films upon AgNPs addition. This reduction could be related to hydrogen bonding or physical crosslinking interactions between AgNPs and the polymer chains, which suppressed water absorption and limited polymer network expansion. In addition, the OTR decreased significantly from 16.17 (PC) to 3.94 (PC_8_), further confirming the positive role of AgNPs in improving the film’s oxygen barrier performance. The well-dispersed AgNPs may have increased the tortuosity of diffusion pathways within the film, thereby effectively impeding the passage of oxygen molecules.

#### 3.2.6. Antimicrobial Activity of Films

**Figure 8:**
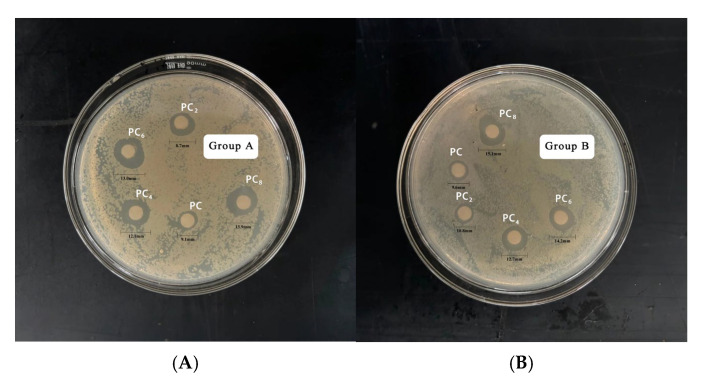
Zone of Inhibition of AgNPs against *E. coli* (**A**); and *S. aureus* (**B**).

Table 5 and Figure 8 shows the antibacterial activity of AgNPs at different concentrations against *E. coli* and *S. aureus*. With increasing AgNPs concentration, the inhibition zone diameter for both bacteria increased significantly, indicating a clear concentration-dependent antibacterial effect. Specifically, as the concentration rose from 0 to 8 mmol/L, the inhibition zone for *E. coli* increased from 9.1 mm to 13.9 mm, and for *S. aureus* from 9.6 mm to 15.1 mm. At all concentrations, AgNPs showed stronger antibacterial activity against *S. aureus* than *E. coli*, suggesting higher sensitivity of Gram-positive bacteria. This may be related to differences in cell wall structure. AgNPs may more easily interact with or penetrate the thick peptidoglycan layer of *S. aureus*, while the outer membrane of *E. coli* may act as a barrier. Notably, when the AgNPs concentration increased from 6 to 8 mmol/L, the growth of inhibition zone diameter slowed, suggesting a plateau effect. This may be due to nanoparticle aggregation or saturation of binding sites on the bacterial surface.

### 3.3. Fruit Preservation

**Figure 9:**
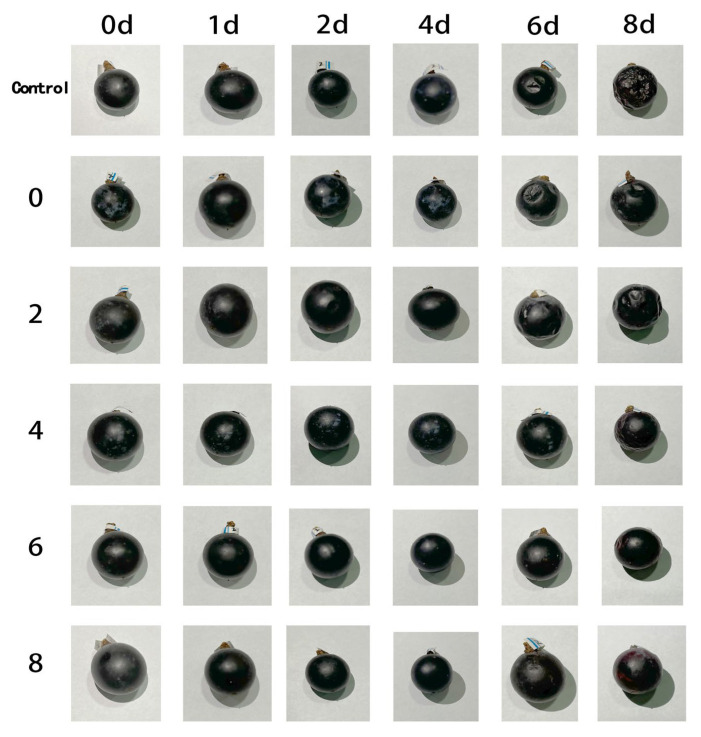
Visual appearance of fruits during storage under different film treatments.

Figure 9 illustrates the visual changes in grape appearance across different treatment groups during an 8-day storage period at ambient temperature. The results show a progressive decline in surface glossiness and the development of varying degrees of skin wrinkling and collapse over time. In the control group, noticeable surface depressions appeared by day 6, and by day 8, the fruit exhibited severe wrinkling and significant moisture loss, leading to a marked deterioration in overall visual quality. In contrast, grapes wrapped with AgNPs/PVA/CS composite films demonstrated superior preservation performance. Notably, the high AgNPs concentration groups (PC_6_ and PC_8_) maintained more intact fruit morphology by the end of storage, with less gloss loss and wrinkling compared to the control. The preservative effect of the films was positively correlated with AgNPs content. These findings suggest that the incorporation of AgNPs effectively slowed moisture loss and tissue degradation in grapes, thereby maintaining sensory quality and structural integrity during storage. This highlights the potential of AgNPs-based composite films for extending the shelf life of fresh produce.

## 4. Conclusions

In this study, grape skin extract (GSE) was successfully employed as a green reductant and stabilizer for the synthesis of silver nanoparticles (AgNPs), which were subsequently incorporated into a PVA/chitosan (CS) matrix to develop a biodegradable and active packaging film. UPLC-MS analysis confirmed that GSE is rich in flavonoids and phenolic acids, such as quercetin and rutin, which facilitated the reduction of Ag^+^ to Ag^0^ and provided effective capping for colloidal stabilization. TEM results revealed that the biosynthesized AgNPs were predominantly spherical, well-dispersed. FTIR and XRD analyses confirmed possible interactions between AgNPs and polymer functional groups through hydrogen bonding and coordination, which influenced both the structural compactness and physical properties of the composite material.

The incorporation of AgNPs significantly enhanced the functional performance of the films. Most notably, the water vapor permeability (WVP) and oxygen transmission rate (OTR) of the PC_8_ film decreased to 43.94 g/(m^2^·24 h) and 3.94 cm^3^/(m^3^·d·Pa), respectively, representing marked improvements in barrier properties. The swelling ratio (SR) also decreased from 67.34% (PC) to 24.30% (PC_8_), suggesting reinforced structural integrity and reduced hydrophilicity. Although the tensile strength and elongation at break decreased at higher AgNPs loadings, which may be attributed to nanoparticle aggregation and disruption of polymer chain interactions, the films still maintained sufficient mechanical integrity for packaging applications. The composite films exhibited strong, dose-dependent antibacterial activity against both *E. coli* and *S. aureus*, with larger inhibition zones observed for *S. aureus*. In practical grape preservation tests, the AgNPs-loaded films effectively delayed dehydration, suppressed microbial growth, and maintained fruit visual quality over 8 days of storage, with the PC_6_ and PC_8_ groups showing the most promising results.

Despite these encouraging outcomes, several limitations should be acknowledged. The composition of GSE is subject to natural variation depending on grape variety, growing conditions, and extraction methods, which may lead to batch-to-batch inconsistencies in AgNPs synthesis. Scaling up the production of GSE-mediated AgNPs and ensuring uniform dispersion in polymer matrices at industrial levels also present challenges that warrant further optimization. Moreover, while the antibacterial and barrier enhancements are clear, the potential migration of AgNPs and their long-term safety in food contact applications require thorough evaluation in future studies.

In summary, this work demonstrates a sustainable and effective strategy for developing bio-based active packaging by valorizing grape processing waste. The GSE-synthesized AgNPs not only impart strong antimicrobial and barrier functionalities but also align with green chemistry principles. With optimized standardization and scale-up protocols, this approach holds considerable potential for commercial application in preserving fresh produce, reducing food waste, and enhancing the sustainability of food packaging systems.

## Figures and Tables

**Table 1:** Abbreviation and ratios of the composite films.

Composite Film	Symbol	Polymer Solids (pva + cs)/g	Glycerol/%	AgNPs/%	AgNPs/g
PVA/CS	PC	6	4	0	0
PVA/CS-2%AgNPs	PC_2_	6	4	2	0.06
PVA/CS-4%AgNPs	PC_4_	6	4	4	0.12
PVA/CS-6%AgNPs	PC_6_	6	4	6	0.18
PVA/CS-8%AgNPs	PC_8_	6	4	8	0.24

**Table 2:** Partial Composition of GSE.

NO.	Name	Retention Time (min)	Area
1	4-Guanidinobutyric acid	0.98	2.05 × 10^4^
2	Gallic acid	1.65	4.09 × 10^3^
3	Parthenolide	9.37	6.93 × 10^3^
4	Nicotinamide	1.29	2.30 × 10^4^
5	lsorhamnetin	12.86	1.90 × 10^4^
6	Hyperoside	1.63	8.86 × 10^4^
7	Genistein	7.82	1.06 × 10^4^
8	Rutin	9.81	2.59 × 10^4^
9	Quercitrin	11.63	8.86 × 10^4^
10	Quercetin	10.24	1.30 × 10^5^
11	Taxifolin	6.30	9.76 × 10^3^
12	Herbacetin-8-glucoside	11.63	8.86 × 10^4^
13	3-Indole-lactic acid	8.38	9.04 × 10^4^
14	Pyroglutamic acid	1.31	3.07 × 10^5^
15	Pyrrolidone carboxylic acid	1.31	3.07 × 10^5^

**Table 3:** Color and transmittance of composite films.

Samples	L*	a*	b*
PC	87.87 ± 0.76 ^a^	1.54 ± 0.39 ^a^	2.34 ± 0.84 ^a^
PC_2_	86.90 ± 1.13 ^a^	1.81 ± 0.58 ^a^	2.67 ± 1.13 ^a^
PC_4_	81.11 ± 2.83 ^b^	6.80 ± 2.72 ^b^	10.91 ± 3.87 ^b^
PC_6_	75.88 ± 2.61 ^c^	9.94 ± 1.16 ^c^	14.21 ± 0.79 ^c^
PC_8_	72.68 ± 1.67 ^d^	13.10 ± 0.84 ^d^	18.65 ± 1.91 ^d^

^a–d^ indicate significant differences (*p* < 0.05) within column L*. ^a–d^ indicate significant differences (*p* < 0.05) within column a*. ^a–d^ indicate significant differences (*p* < 0.05) within column b*.

**Table 4:** WVP, SR and OTR of composite films.

Samples	WVP (g/(m^2^·24 h))	SR (%)	OTR (cm^3^/m^3^.d.Pa)
PC	60.96 ± 0.31 ^a^	67.34 ± 0.52 ^a^	16.17 ± 0.17 ^a^
PC_2_	50.43 ± 0.30 ^b^	55.87 ± 0.94 ^b^	8.04 ± 0.47 ^b^
PC_4_	46.12 ± 0.14 ^c^	40.40 ± 0.54 ^c^	6.5 ± 0.25 ^c^
PC_6_	44.06 ± 0.94 ^d^	36.24 ± 0.49 ^d^	4.61 ± 0.13 ^d^
PC_8_	43.94 ± 0.91 ^d^	24.30 ± 0.49 ^e^	3.94 ± 0.35 ^e^

The letters a to e in the same column indicate significant differences (*p* < 0.05), with ‘a’ representing the greatest difference.

**Table 5:** Diameter of the antibacterial zone of different films.

No.	Concentration of AgNPs(mMol/L)	*E. coli* (mm)	*S. aureus* (mm)
1	0	9.1	9.6
2	2	8.7	10.8
3	4	12.1	12.7
4	6	13.0	14.2
5	8	13.9	15.1

## Data Availability

The original contributions presented in the study are included in the article/Supplementary Materials. Further inquiries can be directed to the corresponding author.

## Supplementary Materials

The following supporting information can be downloaded at: https://www.mdpi.com/article/10.3390/ma19020218/s1, Figure S1: (A) fresh selenium-enriched grapes; (B) grape skin; (C) grape skin powder. Figure S2: Synthesis process of AgNPs.

## Abbreviations

The following abbreviations are used in this manuscript:

AgNPs	Sliver Nanoparticles
GSE	Grapes
PVA	Polyvinyl alcohol
CS	Chitosan
WVP	Water vapor permeability
TS	Tensile strength
EAB	Elongation at break
SR	Swelling ratio
OTR	Oxygen transmission rate
TEM	transmission electron microscopy
FTIR,	Fourier transform infrared spectroscopy
SPR	surface plasmon resonance
*S. aureus*	*Staphylococcus aureus*
*E. coli*	*Escherichia coli*
