# Supplementary material for: A Green Approach to Bio-Based Active Packaging: Grape Skin Extract-Synthesized AgNPs for Food Preservation
Source: Materials (Basel). 2026 Jan 6;19(2):218. doi: 10.3390/ma19020218 (PMC12842933; doi:10.3390/ma19020218)

Supporting information

A Green Approach to Bio-Based Active Packaging: Grape Skin Extract-Synthesized AgNPs for Food Preservation

Wenjia Yin [1], Yongzhen Lei [1,*], Jiayi Wang [1], Qin Lei [2], Wenxi Yu [2] and Siyu Ou [2]

Figure S1. (A) fresh selenium-enriched grapes; (B) grape skin; (C) grape skin powder.
Figure S2. Synthesis process of AgNPs.

Figure S1. (**A**) fresh selenium-enriched grapes; (**B**) grape skin; (**C**) grape skin powder.

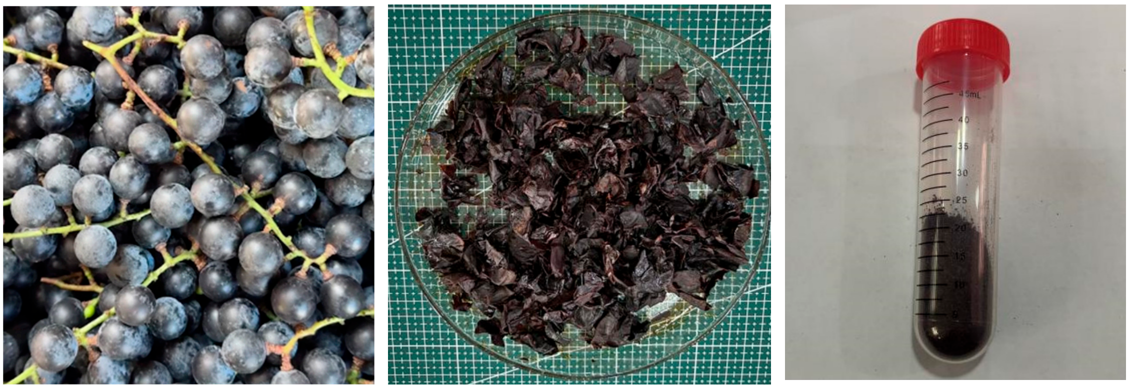

(**A**) (**B**) (**C**)

Figure S2. Synthesis process of AgNPs.

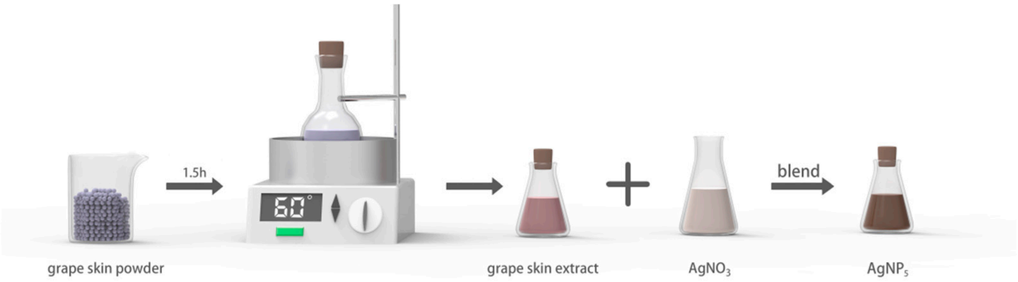

Supplement: Supplementary file 1 [file materials-19-00218-s001.zip › materials-4030047-supplementary.pdf]
